# Cytotoxicity of propofol in human induced pluripotent stem cell-derived cardiomyocytes

**DOI:** 10.1007/s00540-017-2441-0

**Published:** 2017-12-29

**Authors:** Koji Kido, Hiroyuki Ito, Yudai Yamamoto, Koshi Makita, Tokujiro Uchida

**Affiliations:** 1 Department of Anesthesiology, Tokyo Medical and Dental University, Graduate School of Medical and Dental Sciences, 1-5-45 Yushima, Bunkyo-ku, Tokyo, 113-8519 Japan; 2 Division of Anesthesia, Kyoundo Hospital, Tokyo, Japan

**Keywords:** Propofol, Cardiomyocytes, Cytotoxicity, Induced pluripotent stem cells, Mitochondrial dysfunction

## Abstract

**Purpose:**

Propofol infusion syndrome (PRIS) is a lethal condition caused by propofol overdose. Previous studies suggest that pathophysiological mechanisms underlying PRIS involve mitochondrial dysfunction; however, these mechanisms have not been fully elucidated. This study aimed to establish an experimental model of propofol-induced cytotoxicity using cultured human induced pluripotent stem cell (iPSC)-derived cardiomyocytes to determine the mechanisms behind propofol-induced mitochondrial dysfunction, and to evaluate the protective effects of coenzyme Q10 (CoQ10).

**Methods:**

Human iPSC-derived cardiomyocytes were exposed to propofol (0, 2, 10, or 50 µg/ml) with or without 5 µM CoQ10. Mitochondrial function was assessed by measuring intracellular ATP, lactate concentrations in culture media, NAD^+^/NADH ratio, and the mitochondrial membrane potential. Propofol-induced cytotoxicity was evaluated by analysis of cell viability. Expression levels of genes associated with mitochondrial energy metabolism were determined by PCR. Intracellular morphological changes were analyzed by confocal microscopy.

**Results:**

Treatment with 50 µg/ml propofol for 48 h reduced cell viability. High concentrations of propofol (≥ 10 µg/ml) induced mitochondrial dysfunction accompanied by downregulation of gene expression of *PGC*-*1alpha* and its downstream targets (*NDUFS8* and *SDHB*, which are involved in the respiratory chain reaction; and *CPT1B*, which regulates beta-oxidation). Cardiomyocytes co-treated with 5 µM CoQ10 exhibited resistance to propofol-induced toxicity through recovery of gene expression.

**Conclusions:**

Propofol-induced cytotoxicity in human iPSC-derived cardiomyocytes may be associated with mitochondrial dysfunction via downregulation of PGC-1alpha-regulated genes associated with mitochondrial energy metabolism. Co-treatment with CoQ10 protected cardiomyocytes from propofol-induced cytotoxicity.

## Introduction

Propofol infusion syndrome (PRIS) is a rare but lethal condition caused by prolonged propofol overdose [1–3]. The syndrome is characterized by severe metabolic acidosis, cardiac failure, refractory arrhythmia, hyperlipidemia, hepatomegaly accompanied by fatty liver, renal failure, and rhabdomyolysis [4]. The pathophysiological mechanisms of PRIS have been studied using animal models and biochemical assays of tissue samples from patients with PRIS [3, 5–8]. In these studies, the cytotoxicity of propofol was suggested to be associated with intracellular energy metabolism. Exposure to high concentrations of propofol decreased the mitochondrial membrane potential and inhibited mitochondrial respiratory chain complexes. These effects may lead to anaerobic metabolism and reduced ATP production. However, the limited availability of human samples and ethical issues have meant that the mechanism has not been completely elucidated.

Vanlander et al. reported that enzymatic reactions that require coenzyme Q10 (CoQ10) could be targets of inhibition by propofol because of the structural similarity between CoQ10 and propofol [6]. Supplementation of CoQ10 could therefore reduce the propofol-induced decrease in the enzyme activity of mitochondrial respiratory chain complexes. However, their experimental design used a tissue homogenate preparation including mitochondria-enriched fractions. It remains unknown whether CoQ10 can effectively protect cells and tissues from the toxic effects of propofol.

Human induced pluripotent stem cells (iPSCs) are generated from somatic cells by epigenetic reprogramming, and differentiate into various cell types including cardiomyocytes [9]. Compared with experimental models using human tissues or embryonic stem cell lines, models to test drug toxicity using iPSC lines can be easily established with fewer ethical concerns [10–12]. Therefore, we studied propofol-induced cytotoxicity and mitochondrial dysfunction in an experimental model using cultured human iPSC-derived cardiomyocytes to represent cells from one of the main organs affected in PRIS.

We first examined whether some of the pathophysiological changes in PRIS could be reproduced in this cultured human iPSC-derived cardiomyocyte experimental model. We then evaluated propofol-induced mitochondrial dysfunction as a possible mechanism of PRIS in this experimental model. To test our hypothesis that propofol-induced impairment of mitochondrial energy metabolism results from downregulation of the expression of genes associated with respiratory chain complexes, changes in transcription levels of these genes were analyzed using reverse transcription PCR (RT-PCR) after PCR array screening. Finally, we studied the protective effects of CoQ10 against propofol-induced cytotoxicity.

## Methods

### Ethical considerations

All experiments using human iPSC-derived cells were approved by the Institutional Review Board for Use of Human iPSCs at Tokyo Medical and Dental University (approval number 16-005).

### Cell culture

Human iPSC-derived cardiomyocytes were obtained as ReproCardio2 frozen single cell kits (ReproCELL, Kanagawa, Japan). Cells (1 × 10^4^ cells per well in black, clear, flat-bottom 96-well plates (Corning, Corning, NY, USA) unless otherwise specified) were seeded in wells precoated with ReproCoat (ReproCELL) and cultured at 37 °C in a humidified incubator with 95% air and 5% CO_2_ for 8 days.

### Immunocytochemistry

Human iPSC-derived cardiomyocytes (2 × 10^4^ cells per well) were cultured on precoated multi-well glass-bottom 35-mm dishes (Matsunami, Osaka, Japan) for 8 days. After fixation with 4% paraformaldehyde, cells were permeabilized with 0.1% Triton X-100, then blocked with 1% bovine serum albumin (KPL, Gaithersburg, MD, USA) in phosphate-buffered saline, and incubated with primary antibodies for cardiac troponin T (Thermo Fisher Scientific, Waltham, MA, USA) overnight. After washing with phosphate-buffered saline, cells were incubated with the secondary antibody (Alexa Fluor 488 chicken anti-mouse IgG; Invitrogen, Carlsbad, CA, USA) and nuclei were simultaneously counterstained with 4′,6-diamidino-2-phenylindole (Molecular Probes, Carlsbad, CA, USA) for 1 h. Images were acquired with a confocal microscope (TCS SP8; Leica Microsystems, Tokyo, Japan).

### Propofol and coenzyme Q10 treatment

After 8 days of culture, iPSC-derived cardiomyocytes were treated with 0, 2, 10, or 50 µg/ml propofol (Maruishi Pharmaceutical, Osaka, Japan) for 48 h to evaluate the dose-dependent effects of propofol. In each study condition, Intralipos (Otsuka Pharmaceutical Factory, Tokushima, Japan) was added to adjust the triglyceride concentration to 500 µg/ml.

To study the effect of CoQ10 on propofol cytotoxicity, cells treated with propofol (50 µg/ml) were simultaneously incubated with or without 5 µM CoQ10 for 48 h. Aqueous CoQ10 (Nisshin Pharma, Tokyo, Japan), which was emulsified to create approximately 50-nm nanoparticles, was used. Half of the culture media containing the treatment solution was changed every 24 h, and assays were conducted after 48 h of treatment.

### Cellular ATP levels assay

Intracellular energy balance was evaluated using a mitochondrial ToxGlo assay (Promega). According to the manufacturer’s protocol, an ATP detection reagent was added to the cells, and ATP levels were determined by measuring the luminescence generated, which was proportional to the amount of ATP.

### Cell viability and caspase 3/7 activity assay

Cell viability and caspase 3/7 activity assays was conducted using an ApoTox-Glo Triplex Assay kit (Promega, Madison, WI, USA). Cell viability, determined by live-cell protease activity, was assessed by measuring fluorescence. Caspase 3/7 activity was analyzed by measuring luminescence with a GloMax microplate reader (Promega) according to the manufacturer’s protocol.

### Measurement of lactate concentrations in culture media

To evaluate changes in metabolic states, lactate concentrations in culture media were quantified using a colorimetric l-lactate assay kit (Abcam, Cambridge, UK). Lactate concentrations in cell culture supernatants were assessed by measuring the optical density at 570 nm using an iMark microplate reader (Bio-Rad Laboratories, Hercules, CA, USA) according to the manufacturer’s protocol.

### NAD^+^/NADH ratio assay

Changes in the NAD^+^/NADH ratio were evaluated using an NAD^+^/NADH-Glo assay kit (Promega). NAD^+^ (oxidized NAD) and NADH (reduced NAD) levels were individually assessed in lysate of cardiomyocytes cultured in the same well by measuring luminescence according to the manufacturer’s protocol.

### Mitochondrial membrane potential assay

To evaluate mitochondrial activity, the mitochondrial membrane potential was assessed using a Mito-ID membrane potential cytotoxicity kit (Enzo Life Sciences, Farmingdale, NY, USA). The mitochondrial membrane potential was analyzed by measuring fluorescence in accordance with the manufacturer’s protocol. Carbonyl cyanide 3-chlorophenylhydrazone (CCCP), which induces mitochondrial depolarization, was used as a positive control. CCCP was added to a final concentration of 4 µM for 30 min before analysis. The mitochondrial membrane potential in each experimental condition was calculated by subtracting the fluorescence of cardiomyocytes treated with CCCP from the fluorescence measured in each sample.

### Mitochondrial morphologic assessment

Human iPSC-derived cardiomyocytes were cultured in multi-well glass-bottom dishes (2 × 10^4^ cells per well) and exposed to propofol or control conditions. To label mitochondria, live cells were incubated with 100 nM MitoTracker Red CMXRos probes (Invitrogen). Additionally, cardiac troponin T and nuclei were counterstained after fixation, as described above. Images were obtained using a TCS SP8 confocal microscope (Leica Microsystems).

### RNA extraction

Human iPSC-derived cardiomyocytes were cultured in 96-well plates. Total RNA was extracted from cells from eight wells (8 × 10^4^ cells) per treatment condition using an RNeasy Micro kit (Qiagen, Valencia, CA, USA) and purified with DNase (Qiagen) to eliminate genomic DNA, following the manufacturer’s protocol. Total RNA was quantified using a Qubit fluorometer (Invitrogen).

### Analyses for mRNA expression by reverse transcription PCR

To screen for possible changes in mRNA expression that might cause mitochondrial dysfunction, an RT^2^ Profiler PCR Array Human Mitochondrial Energy Metabolism kit (PAHS-008Z; Qiagen) was used. RNA (100 ng) from each sample was reverse transcribed to cDNA, which was then preamplified using an RT^2^ PreAMP cDNA synthesis kit and RT^2^ PreAMP Pathway primer mix (Qiagen). A PCR mix comprising preamplified cDNA, SYBR Green Master Mix (Qiagen), DNase-free water, and primers for each target mRNA was added to the 96-well plate (25 µl per well), and PCR was performed using a LightCycler 96 (Roche Diagnostics GmbH, Mannheim, Germany). Relative expression levels were analyzed and compared statistically with Qiagen web-based software for RT^2^ Profiler PCR array (http://pcrdataanalysis.sabiosciences.com/pcr/arrayanalysis.php).

To confirm the results of the PCR array, expression levels of screened mRNA were verified individually by quantitative RT-PCR (qRT-PCR). Additional genes associated with mitochondrial function and their regulators were also studied. cDNA was prepared from RNA (500 ng) from each sample using a First Strand cDNA synthesis kit (Roche Diagnostics GmbH). The sequences of the primers used for PCR are presented in Table 1. A mixture of cDNA, primers, FastStart Essential DNA Green Master (Roche Diagnostics GmbH), and DNase-free water was added to 96-well plates (20 µl per well), and real-time qRT-PCR was performed using a LightCycler 96. A melting curve analysis was also run. Relative expression levels were normalized to expression of a housekeeping gene, beta-actin (*ACTB*), using the ΔΔ*C*_q_ method.

**Table 1:** Primer sequences for real-time RT-PCR

Gene	Primer	Sequence (5′–3′)
*ACTB*	Left	ccaaccgcgagaagatga
	Right	ccagaggcgtacagggatag
*NDUFS8*	Left	tgcttggcaaggcaagtag
	Right	tctcgggatcctgcatgt
*SDHB*	Left	ggggcctgcagttcttatg
	Right	aggcgctcctctgtgaagt
*PGC*-*1alpha*	Left	tgagagggccaagcaaag
	Right	ataaatcacacggcgctctt
*NRF1*	Left	ccatctggtggcctgaag
	Right	gtagtgcctgggtccatga
*NRF2*	Left	ggacgggtctaggtgagaca
	Right	tggctggagtatttcaaaggat
*PDSS1*	Left	agatctggggtgaaaagaagg
	Right	ccaattcgtgccagagcta
*PPARalpha*	Left	gcactggaactggatgacag
	Right	tttagaaggccaggacgatct
*CPT1B*	Left	tgtgagtgactggtgggaag
	Right	ttgatgagcacaaggtccat

*ACTB* beta-actin, *NDUFS8* NADH:ubiquinone oxidoreductase core subunit S8, *SDHB* succinate dehydrogenase complex iron sulfur subunit B, *PGC*-*1alpha* peroxisome proliferator-activated receptor gamma coactivator 1 alpha, *NRF1* nuclear respiratory factor 1, *NRF2* nuclear respiratory factor 2, *PDSS1* decaprenyl diphosphate synthase subunit 1, *PPARalpha* peroxisome proliferator-activated receptor alpha, *CPT1B* carnitine palmitoyltransferase 1B

### Statistical analysis

Data were obtained from three independent samples in each condition in PCR array screening, four or five independent samples in followed qRT-PCR experiments, and four independent samples in other experiments. Values are presented as individual raw data. Statistical significance was assessed using the Mann–Whitney *U* test to analyze differences of non-parametric data between two groups, and the Steel’s multiple comparisons test to analyze them between control and other experimental conditions. Analyses were conducted using Stata/IC software (ver. 13; StataCorp, College Station, TX, USA). A *P* value < 0.05 was considered statistically significant.

## Results

### Induced pluripotent stem cell-derived cardiomyocyte phenotypes and responses to toxic effects of high concentrations of propofol

**Fig. 1:**
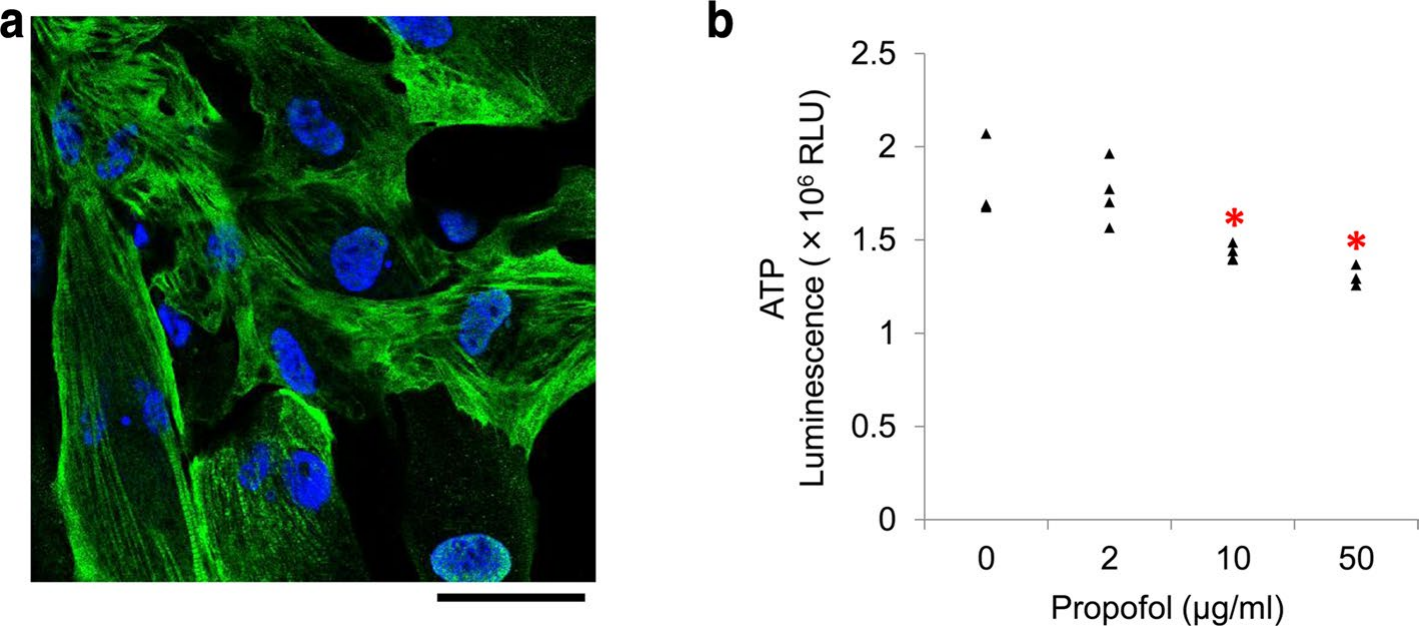
Cultured human induced pluripotent stem cell (iPSC)-derived cardiomyocytes as an experimental model to study propofol cytotoxicity. **a** Immunostaining of human iPSC-derived cardiomyocytes. Confocal microscopy showing human iPSC-derived cardiomyocytes stained positive for cardiac troponin T (*green*) 8 days after seeding. Nuclei were counterstained with 4′,6-diamidino-2-phenylindole (DAPI,* blue*).* Scale bar*, 50 μm. **b** Propofol-induced reduction in ATP levels in human iPSC-derived cardiomyocytes. Treatment with ≥ 10 µg/ml propofol for 48 h significantly reduced intracellular ATP. Data are presented as* triangular dots*; *n* = 4 in each experimental condition. *P < 0.05, compared with control conditions (0 µg/ml propofol in triglyceride emulsion). *RLU* relative luminescence units

Immunostaining showed that human iPSC-derived cardiomyocytes contained the cardiac-specific marker cardiac troponin T after 8 days of culture (Fig. 1a). When cardiomyocytes were cultured to form a cell clump, they started to contract 3 or 4 days after seeding, as visualized by optical microscopy.

Cellular ATP levels significantly decreased with treatment with ≥ 10 µg/ml propofol for 48 h (Fig. 1b). Further, 50 µg/ml propofol resulted in a significant elevation the lactate concentration in the culture media (*P* = 0.02), and reduction in cell viability (*P* = 0.04) compared with cells treated with a propofol-free triglyceride emulsion. To study a possible cause of reduced cell viability, we measured caspase 3/7 activity as a marker of apoptosis. However, there were no differences among cells treated with each condition (data are not shown).

### Concentration-dependent induction of mitochondrial dysfunction by propofol

**Fig. 2:**
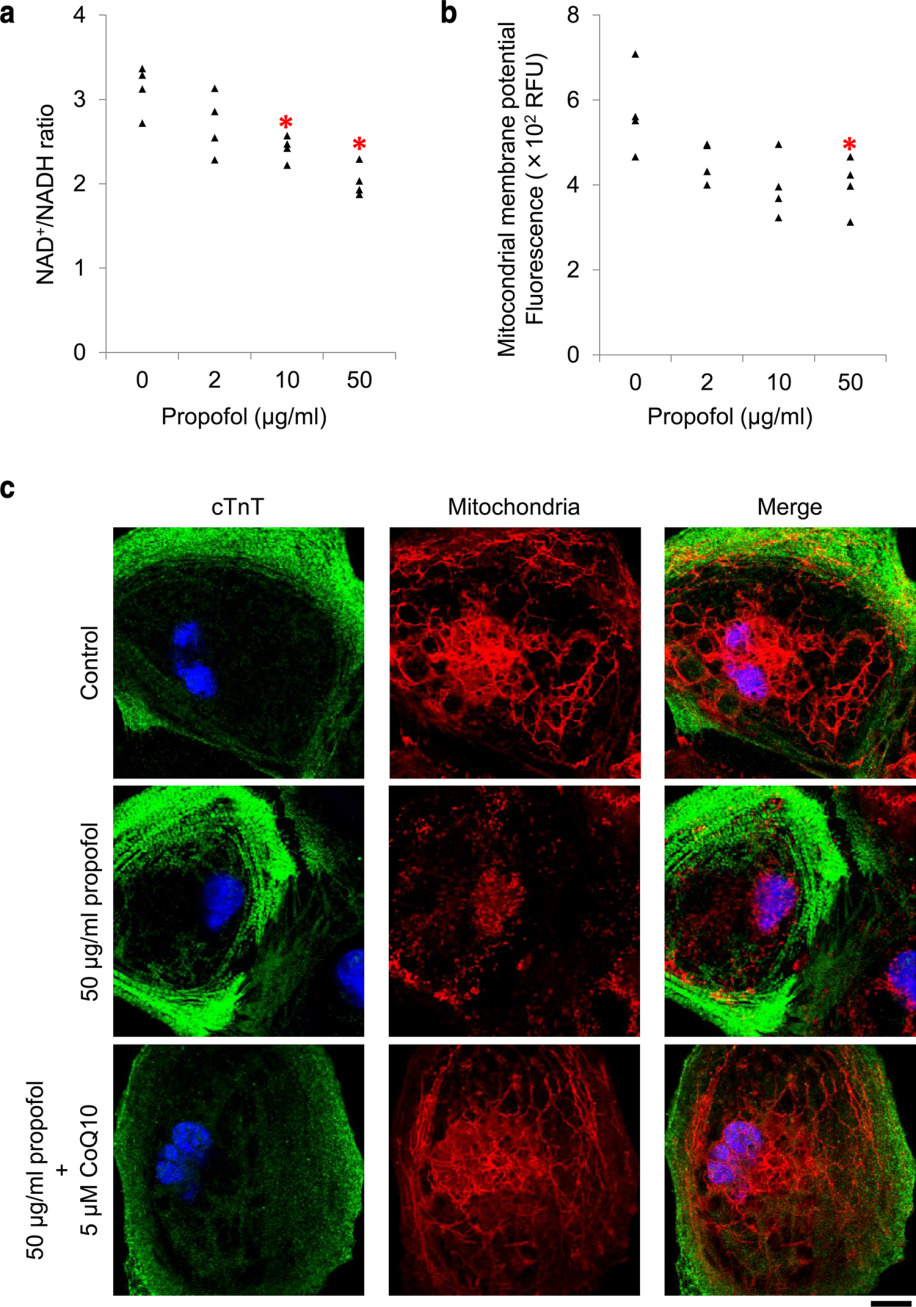
Mitochondrial dysfunction caused by propofol treatment at high concentrations for 48 h. **a** Effect of propofol on NAD^+^/NADH ratio in human induced pluripotent stem cell (iPSC)-derived cardiomyocytes. NAD^+^/NADH ratio was significantly reduced by 48 h of propofol exposure (10 or 50 µg/ml) compared with control conditions, suggesting disruption of the H^+^/electron transport system and NADH accumulation. **b** Quantification of the mitochondrial membrane potential in propofol-treated cardiomyocytes. Treatment with 50 µg/ml propofol for 48 h significantly reduced the mitochondrial membrane potential. Carbonyl cyanide 3-chlorophenylhydrazone (CCCP), which induces mitochondrial depolarization, was used as a positive control. Data were normalized by subtracting the fluorescence of cardiomyocytes treated with 4 μM CCCP from each measurement. **c** Morphologic changes in mitochondria of iPSC-derived cardiomyocytes after treatment with propofol, visualized using confocal microscopy. After exposure to each condition for 48 h, cardiomyocytes were stained for cardiac troponin T (cTnT,* green*), and with MitoTracker Red CMXRos (mitochondria,* red*) and 4′,6-diamidino-2-phenylindole (DAPI, nuclei,* blue*).* Scale bar*, 10 μm. Data are presented as* triangular dots*; *n* = 4 in each experimental condition. *P < 0.05, compared with control conditions. *RFU* relative fluorescence units

To investigate the mechanisms underlying the propofol-induced reduction in ATP levels, we evaluated the function of mitochondria, the major source of ATP. As shown in Fig. 2a, propofol reduced the NAD^+^/NADH ratio in a concentration-dependent manner, which suggested H^+^/electron transport system dysfunction and NADH accumulation. Next, we analyzed the mitochondrial membrane potential and found that it was reduced with 50 µg/ml propofol (Fig. 2b, *P* = 0.04). Consistent with this quantification, confocal microscopy of cultured cardiomyocytes stained with an activated mitochondrial marker (MitoTracker) showed that cells treated with 50 µg/ml propofol exhibited shortened or fragmented mitochondria, whereas cells cultured in control conditions had long, filamentous mitochondria (Fig. 2c).

**Fig. 3:**
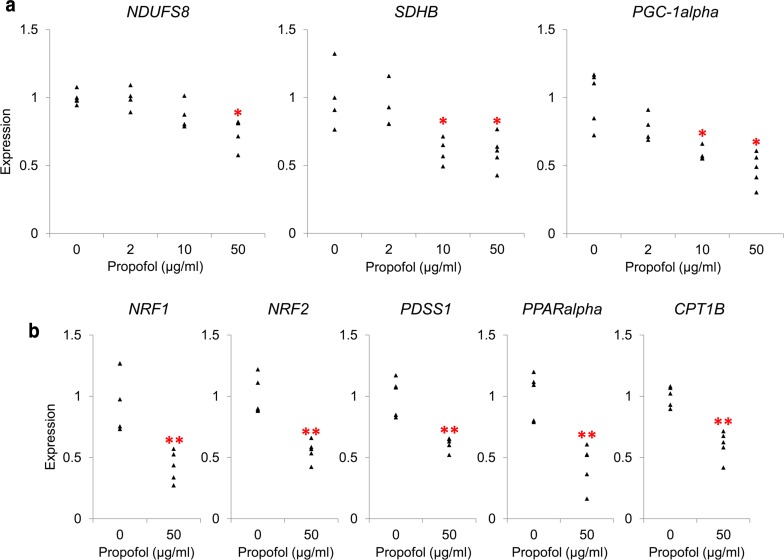
Propofol-induced downregulation of genes regulating mitochondrial energy metabolism. **a** Effect of propofol on expression of genes regulating mitochondrial function. qRT-PCR demonstrated that treatment with propofol for 48 h downregulated *NDUFS8* (complex I,* left*, at 50 µg/ml) and *SDHB* (complex II,* middle*, ≥ 10 µg/ml). Expression of *PGC*-*1alpha* (an upstream regulator of mitochondrial function) was also reduced with ≥ 10 µg/ml propofol (*right*). **b** Propofol-induced downregulation of downstream genes regulated by *PGC*-*1alpha*. Downstream target genes of *PGC*-*1alpha*, specifically *NRF1/2* (which encodes a regulator of mitochondrial complex genes), *PDSS1* (which encodes a coenzyme Q10 [CoQ10] synthesis enzyme), *PPARalpha,* and *CPT1B* (which encodes a regulator of beta oxidation) were downregulated by treatment with 50 µg/ml propofol. Each transcript level was normalized to beta-actin (*ACTB*). Data are presented as* triangular dots*; *n* = 4–5 in each experimental condition. *P < 0.05, **P < 0.01, compared with control conditions (0 µg/ml propofol in triglyceride emulsion)

To further analyze mitochondrial function, we conducted a PCR array of 84 genes using an RT^2^ Profiler PCR array Human Mitochondrial Energy Metabolism kit. Propofol (50 µg/ml) reduced the expression of transcripts of genes associated with mitochondrial complexes I–V (Table 2). To verify the dose-dependence of propofol-induced downregulation, we examined the expression of *NDUFS8* (which encodes NADH:ubiquinone oxidoreductase core subunit S8, a component of complex I) and *SDHB* (which encodes succinate dehydrogenase complex iron sulfur subunit B, of complex II), again using real-time qRT-PCR. Both genes were downregulated with 50 µg/ml propofol (Fig. 3a, left and middle, *P* = 0.01 for NDUFS8, and *P* = 0.02 for SDHB).

**Table 2:** Mitochondrial energy metabolism PCR array showing genes exhibiting propofol-induced downregulation

Gene	Expression ratio (vs. control)	95% CI
Complex I		
*NDUFB2*	0.83	0.69, 0.96
*NDUFB3*	0.82	0.69, 0.94
*NDUFB4*	0.80	0.67, 0.92
*NDUFB9*	0.84	0.72, 0.95
*NDUFB10*	0.85	0.72, 0.98
*NDUFS3*	0.80	0.63, 0.97
*NDUFS8*	0.59	0.47, 0.71
*NDUFV2*	0.84	0.75, 0.94
*NDUFV3*	0.63	0.46, 0.80
Complex II		
*SDHB*	0.83	0.74, 0.92
*SDHD*	0.48	0.22, 0.74
Complex III		
*UQCRC1*	0.81	0.64, 0.98
*UQCRC2*	0.84	0.69, 0.99
*UQCRFS1*	0.86	0.75, 0.97
Complex IV		
*COX4I1*	0.65	0.45, 0.85
*COX4I2*	0.59	0.26, 0.91
*COX8C*	0.82	0.67, 0.96
Complex V		
*ATP5G1*	0.81	0.66, 0.96
*ATP6V0D2*	0.30	0.11, 0.48
*OXA1L*	0.76	0.64, 0.88

Propofol (50 µg/ml) reduced the expression of transcripts of genes associated with mitochondrial complexes. Relative expression levels were analyzed and compared statistically with Qiagen web-based software for RT2 Profiler PCR array. Data are mean and confidence interval (CI); *n* = 3 in each experimental condition

*NDUFB2* NADH:ubiquinone oxidoreductase subunit B2, *NDUFB3* NADH:ubiquinone oxidoreductase subunit B3, *NDUFB4* NADH:ubiquinone oxidoreductase subunit B4, *NDUFB9* NADH:ubiquinone oxidoreductase subunit B9, *NDUFB10* NADH:ubiquinone oxidoreductase subunit B10, *NDUFS3* NADH:ubiquinone oxidoreductase core subunit S3, *NDUFS8* NADH:ubiquinone oxidoreductase core subunit S8, *NDUFV2* NADH:ubiquinone oxidoreductase core subunit V2, *NDUFV3* NADH:ubiquinone oxidoreductase core subunit V3, *SDHB* succinate dehydrogenase complex iron sulfur subunit B, *SDHD* succinate dehydrogenase complex subunit D, *COX4I1* cytochrome c oxidase subunit 4I1, *COX4I2* cytochrome c oxidase subunit 4I2, *COX8C* cytochrome c oxidase subunit 8C, *ATP5G1* ATP synthase, H^+^ transporting, mitochondrial Fo complex subunit C1 (subunit 9), *ATP6V0D2* ATPase H^+^ transporting V0 subunit d2, *OXA1L* OXA1L, mitochondrial inner-membrane protein

Because these genes are regulated by peroxisome proliferator-activated receptor gamma coactivator 1 alpha (PGC-1alpha) [13], we investigated whether *PGC*-*1alpha* was affected by propofol. We found that propofol dose-dependently reduced *PGC*-*1alpha* expression; the reduction was statistically significant in experimental conditions with ≥ 10 µg/ml propofol (Fig. 3a, right). Propofol also decreased the expression of downstream target genes of *PGC*-*1alpha*: nuclear respiratory factor 1/2 (*NRF1/2*), a mitochondrial gene regulatory gene; decaprenyl diphosphate synthase subunit 1 (*PDSS1*), a gene encoding an enzyme involved in CoQ10 synthesis; peroxisome proliferator-activated receptor alpha (*PPARalpha*); and carnitine palmitoyltransferase 1B (*CPT1B*), a beta-oxidation regulatory gene (Fig. 3b).

### Coenzyme Q10 treatment protected against the toxic effects of propofol in cultured cardiomyocytes

**Fig. 4:**
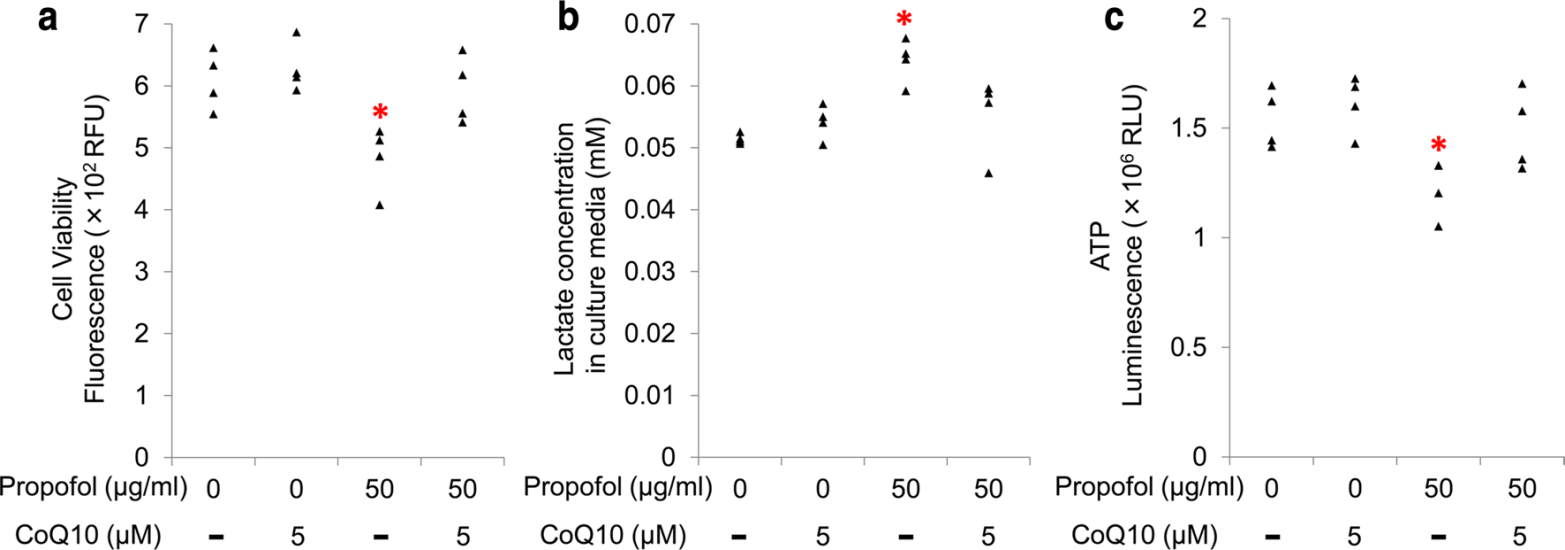
Protective effects of coenzyme Q10 (CoQ10) against propofol cytotoxicity in human induced pluripotent stem cell (iPSC)-derived cardiomyocytes. iPSC-derived cardiomyocytes were co-treated with CoQ10 and 50 µg/ml propofol for 48 h. Supplementation of 5 µM CoQ10 protected cardiomyocytes from propofol-induced reduction of cell viability (**a**), elevation of lactate concentrations in culture media (**b**), and reduction in cellular ATP levels (**c**). Data are presented as* triangular dots*;* n* = 4 in each experimental condition. *P < 0.05, compared with control conditions (0 µg/ml propofol in triglyceride emulsion). *RFU* relative fluorescence units, *RLU* relative luminescence units

**Fig. 5:**
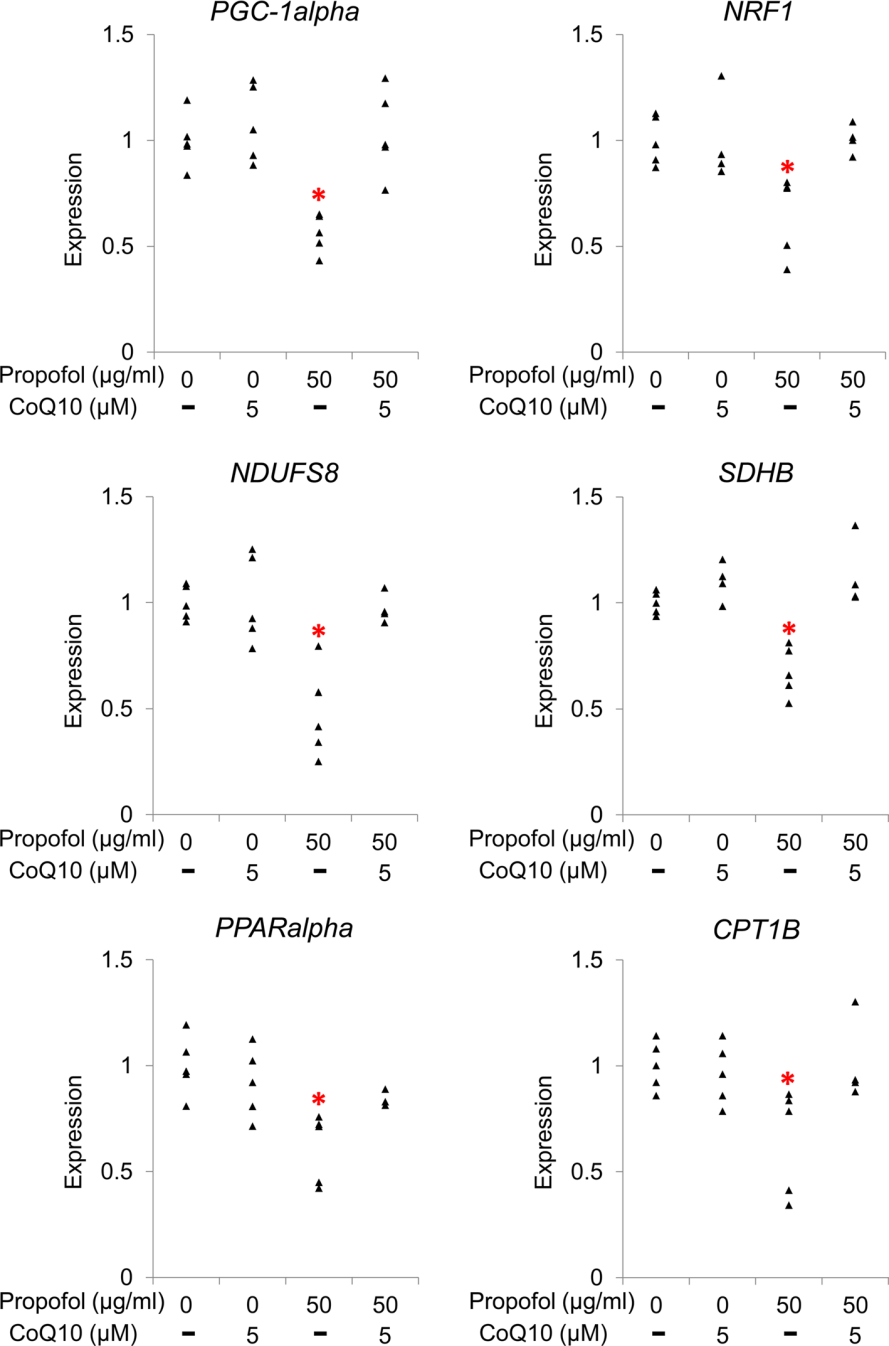
Effect of coenzyme Q10 (CoQ10) on propofol-induced downregulation of genes regulating mitochondrial energy metabolism. Co-treatment with CoQ10 (5 µM) protected cardiomyocytes from propofol-induced (50 µg/ml for 48 h) reduction in gene expression of *PGC*-*1alpha*, *NRF1*, *NDUFS8*, *SDHB*, *PPARalpha*, and *CPT1B*. Transcript levels were normalized to *ACTB*. Data are presented as* triangular dots*; *n* = 4–5 in each experimental condition. *P < 0.05, compared with control conditions (0 µg/ml propofol in triglyceride emulsion)

Because of the structural similarities between propofol and CoQ10, studies have hypothesized that propofol interferes with respiration [6, 7]. We added CoQ10 to the culture media during propofol exposure and investigated whether this treatment protected against the toxic effects of propofol in cardiomyocytes, which includes mitochondrial dysfunction. We used emulsified CoQ10 at 5 µM because of its stability in aqueous solution. When CoQ10 was added to the culture media, propofol (50 µg/ml) did not reduce cell viability, increase lactate production, or reduce intracellular ATP levels (Fig. 4a–c). Consistent with these findings, confocal microscopy showed that CoQ10 treatment reduced propofol-induced morphological changes in mitochondria associated with mitochondrial dysfunction (Fig. 2c). CoQ10 treatment also prevented propofol-induced reduction in the expression of *PGC*-*1alpha*, *NRF1*, *NDUFS8*, *SDHB*, *PPARalpha*, and *CPT1B* (Fig. 5).

## Discussion

In this study, propofol-induced cytotoxicity was reproduced in an experimental model using cultured human iPSC-derived cardiomyocytes. Treatment with high concentrations of propofol (≥ 10 µg/ml) for 48 h significantly impaired mitochondrial function, and 50 µg/ml propofol reduced cell viability and elevated lactate concentrations in culture media. Mitochondrial dysfunction could at least partially result from downregulation of gene expression of *PGC*-*1alpha* and associated genes that regulate mitochondrial energy metabolism. CoQ10 treatment of propofol-exposed cells rescued mitochondrial function and cell viability through recovery of gene expression.

### Cultured induced pluripotent stem cell-derived cardiomyocytes as an experimental model for investigating propofol toxicity

Because of the need for high-throughput assays for human drug toxicity and the relatively minimal associated ethical issues, experimental models using cultured iPSC-derived cardiomyocytes have recently been developed and applied in the field of drug safety testing, especially for drug-induced arrhythmia [14–16]. iPSC-derived cardiomyocytes have characteristically similar ion channels, receptors, transporters, and contractile activity as native cardiac tissue or cells [17, 18]. There is, however, debate about the similarities in mitochondrial function between native and iPSC-derived cardiomyocytes. A study reported that genes associated with mitochondrial energy metabolism in iPSC-derived cardiomyocytes showed lower expression levels than native cardiac tissue; nevertheless, these cells had potential as a screening model for drug-induced mitochondrial toxicity [19]. iPSC-derived cardiomyocytes were used to study the cardioprotective effects of isoflurane under oxidative stress conditions by evaluating mitochondrial morphology and function [20, 21]. In this context, iPSC-derived cardiomyocytes could be used for evaluation of mitochondrial function.

In the current study, we used commercially available human iPSC-derived cardiomyocytes, which exhibit a cardiomyocyte phenotype, to examine the toxicity of high concentrations of propofol, because cardiomyocytes are one of the main cell types affected in PRIS. We found that ≥ 10 µg/ml propofol caused mitochondrial dysfunction, and 50 µg/ml propofol induced cell death and elevated lactate concentrations in culture media. These findings are consistent with the clinical signs of PRIS. There are no reports showing plasma or tissue concentrations of propofol in PRIS patients. Because the infusion of propofol at 4 mg/kg/h for a 48-h duration is considered the upper limit for the prevention of PRIS [2, 4], a relatively safe plasma concentration of approximately 2.5 μg/ml could be predicted using the Marsh model [22]. The toxic range of propofol in the current experimental model (≥ 10 µg/ml) is much higher than the plasma concentration range observed under normal clinical conditions, and is consistent with concentrations observed in cases of propofol overdose, and the toxicity was concentration-dependent. The pathophysiological changes associated with PRIS could be reproduced in our experimental model using cultured iPSC-derived cardiomyocytes.

Because previous studies have reported apoptosis in cultured cells treated with propofol [5, 23], we investigated caspase 3/7 activity as a measure of apoptosis. We observed no change in caspase 3/7 activity in cardiomyocytes treated with propofol in the experimental model. Although we could not exclude the possibility of the toxic effects of propofol contributing to apoptosis, the current focus of the study was the regulation of mitochondrial function in the experimental model using iPSC-derived cardiomyocytes. Nevertheless, our current results need to be validated using in vivo or ex vivo experimental models as a next step.

### Mechanisms of propofol-induced mitochondrial dysfunction

Impaired mitochondrial function is thought to be one of the mechanisms underlying PRIS. This has mostly been demonstrated in cell-free models using isolated mitochondria [3, 6, 7]. The results of the current study show that ≥ 10 µg/ml propofol reduced the intracellular NAD^+^/NADH ratio, which reflects disruption of electron transport. At the same range of propofol concentrations, the mitochondrial membrane potential was reduced, and fragmented mitochondria were observed by confocal microscopy. These changes in mitochondria reflect the reduction in intracellular energy production and activation of anaerobic metabolism that were indicated by the decrease in intracellular ATP levels and the increase in lactate concentrations in culture media.

To study the mechanisms for propofol-induced mitochondrial dysfunction, we evaluated the expression of genes associated with mitochondrial energy metabolism. PCR array analysis revealed that exposure to high concentrations of propofol induced alterations in the expression of a number of genes that encode components of the mitochondrial respiratory chain complexes. *NDUFS8* (which encodes a subunit of complex I) and *SDHB* (complex II) were downregulated with high concentrations of propofol, in keeping with the mitochondrial function quantification results. It is reported that expression of these mitochondrial respiratory chain components is regulated by an upstream regulator, PGC-1alpha, which is also a prominent transcriptional coactivator that regulates the production of CoQ10 via NRF1/2, and fatty acid beta-oxidation via PPARalpha [13, 24–27]. Beta-oxidation has been suggested as another target of the inhibitory effects of propofol in PRIS [3]. Therefore, we explored the expression of *PGC*-*1alpha* and its downstream target genes. Our results indicated that *PGC*-*1alpha* expression significantly decreased with propofol, and downregulation of *NRF1/2*, *PPAR*-*alpha*, *PDSS1* (which encodes an enzyme involved in CoQ10 production), and *CPT1B* (which encodes a rate-limiting enzyme in beta-oxidation) were observed. There are several possibilities for this negative regulation of *PGC*-*1alpha* by propofol. First, given that previous studies have reported that CoQ10, which is structurally similar to propofol, can increase PGC-1alpha expression [28–30], propofol may act as a competitive inhibitor of *PGC*-*1alpha* expression. Second, several reports suggest that propofol inactivates Akt [5, 31], and that Akt activity regulates *PGC*-*1alpha* expression [32, 33]; therefore, this Akt-mediated regulatory mechanism may also explain propofol-induced downregulation of *PGC*-*1alpha*. Mitochondrial impairment caused by downregulated *PGC*-*1alpha* has been suggested as a mechanism of several pathological conditions including infectious diseases [28, 34, 35], lipid overload disease [32, 36], and statin-induced myopathy [29]. Suppression of the *PGC*-*1alpha*-mediated pathway could be involved in propofol-induced mitochondrial dysfunction.

### Coenzyme Q10 protects against propofol-induced mitochondrial dysfunction

**Fig. 6:**
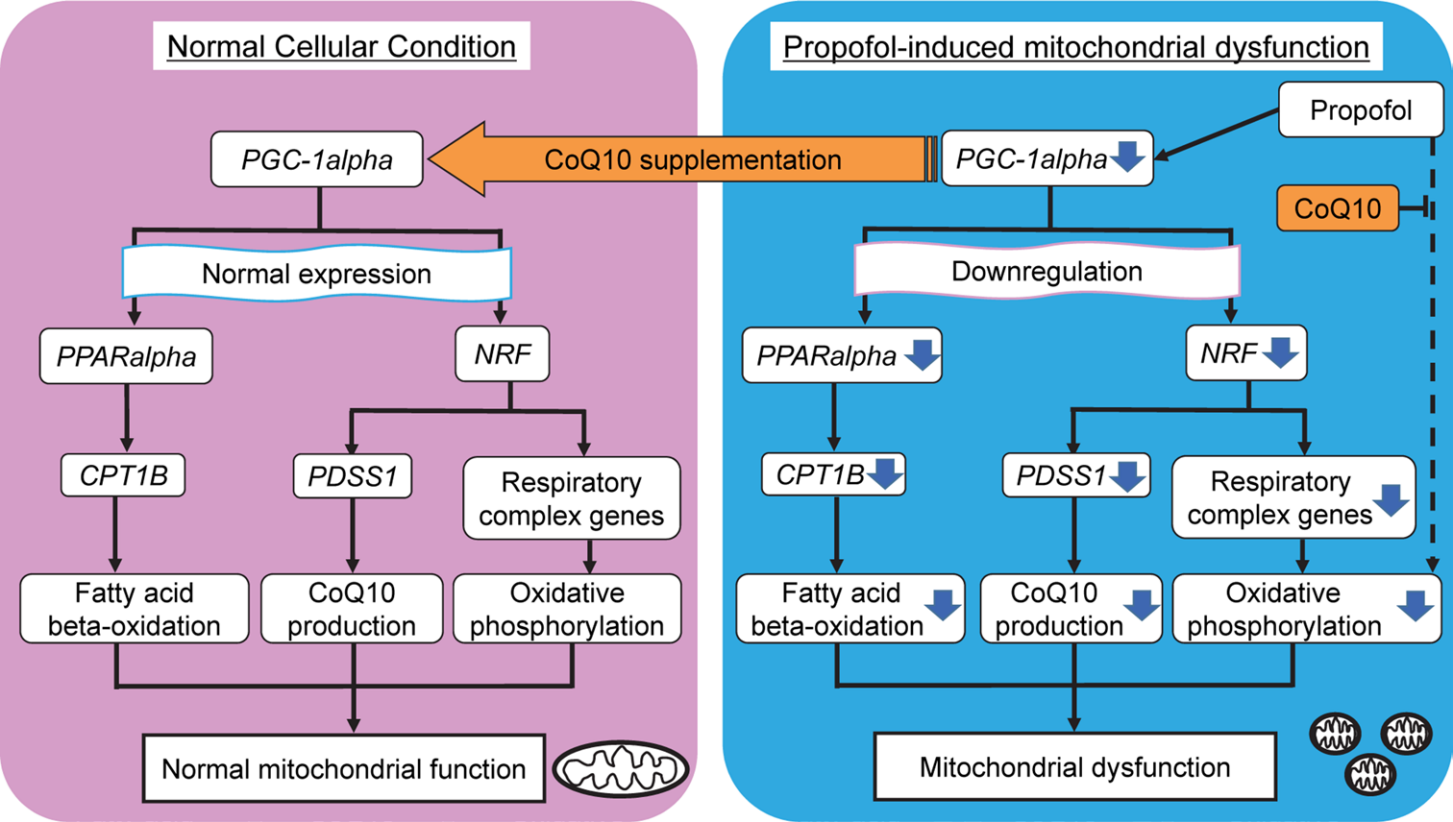
Model of propofol-induced mitochondrial dysfunction and protective effects of coenzyme Q10 (CoQ10). Mitochondrial dysfunction can be caused by propofol-induced downregulation of PGC-1-alpha and its downstream target genes associated with mitochondrial energy metabolism in cardiomyocytes. As an additional mechanism, propofol can take over the role of CoQ10 and inhibit oxidative phosphorylation directly in mitochondria. Co-treatment with CoQ10 can contribute to the maintenance of mitochondrial function through recovery of gene expression

Vanlander et al. reported that CoQ10, which is structurally similar to propofol, ameliorates the decreased enzyme activity observed in complex II and III in skeletal muscle homogenates exposed to propofol [6]. Bergamini et al. found that CoQ10 supplementation reduced propofol-induced mitochondrial damage and cytotoxicity in cell culture models using human cell lines [7]. These reports focused on the direct inhibitory effects of propofol on oxidative phosphorylation in mitochondria. In the current study, we found that cardiomyocytes treated with 5 µM CoQ10 were protected against propofol-induced mitochondrial dysfunction and cell death. Additionally, we found that expression of *PGC*-*1alpha* and associated genes was not affected by propofol in cells treated with CoQ10. These protective effects of CoQ10 might contribute to the maintenance of mitochondrial function in human cardiomyocytes (Fig. 6). Our results suggest that extrinsic administration of CoQ10 might have potential as a preventative treatment for propofol-induced toxicity, at least as a factor to ameliorate mitochondrial function and related metabolic reactions.

### Study limitations

This study has some limitations. First, the study was designed as a cell culture experiment, and the results cannot be directly extrapolated to the responses of human tissues to high doses of propofol. For example, the results simply showed cytotoxicity of propofol under an oxygen concentration higher than that in peripheral tissue. Hypoxic conditions per se could cause cellular damage and could modify the toxicity of high-dose propofol. In this context, further studies are needed to determine factors that can be modified in propofol-induced cytotoxicity, including the oxygen concentration. Second, this study was designed principally to screen molecular changes in the cytotoxic mechanism of propofol, and the size of the study was small. Further ex vivo or in vivo studies are required to confirm the findings, and the calculation of an appropriate sample size using power analysis based on our current results is needed. Third, although we demonstrated propofol-induced transcriptional downregulation of *PGC*-*1alpha*, post-transcriptional regulation was not analyzed because of difficulties in the preparation of protein samples for such analyses. However, the reduced expression of several genes regulated by PGC-1alpha strongly supports a reduction in *PGC*-*1alpha* gene expression associated with this negative regulation. Fourth, although we focused on changes in gene expression in this study, we cannot exclude contributions from other mechanisms including the Akt–GSK-3 pathway [5], microRNA [31], and mitochondrial DNA abnormalities [37, 38]. Neither could we show whether propofol and CoQ10 directly affected respiratory chain activity, or whether propofol and CoQ10 were incorporated into mitochondria. In this context, further studies using isolated mitochondria from human tissue or iPSC derived cardiomyocyte from large scale culture are needed to prove the hypothesis derived from studies using isolated mitochondria derived from animal tissue [6, 7]. Both propofol and CoQ10 in the current study were emulsified for stability in water. This could be a helpful method for minimizing differences in the distribution of propofol and CoQ10. We demonstrated a competitive relationship between these two molecules, and multiple mechanisms might contribute to the mode of action. Finally, we did not study the effect of proposed clinical aggravating factors, including catecholamines and steroids [4]. However, the current iPSC-derived cell culture model may be useful to study whether these factors enhance propofol toxicity.

## Conclusions

We established an experimental model reproducing propofol-induced cytotoxicity using human iPSC-derived cardiomyocytes. Treatment with propofol (≥ 10 µg/ml) for 48 h impaired mitochondrial function via downregulation of expression of *PGC*-*1alpha*-regulated genes associated with mitochondrial energy metabolism. These effects may reduce cell viability. Furthermore, CoQ10 could play an important role in conferring resistance to propofol cytotoxicity by restoring *PGC*-*1alpha* expression. Supplementation of CoQ10 could therefore be used as a prophylactic or therapeutic approach to propofol-induced toxicity.
